# A Pilot Study of EEG Source Analysis Based Repetitive Transcranial Magnetic Stimulation for the Treatment of Tinnitus

**DOI:** 10.1371/journal.pone.0139622

**Published:** 2015-10-02

**Authors:** Hui Wang, Bei Li, Yanmei Feng, Biao Cui, Hongmin Wu, Haibo Shi, Shankai Yin

**Affiliations:** Department of Otolaryngology head and neck surgery, Shanghai Jiao Tong University Affiliated Sixth People’s Hospital, Shanghai, China, 200233; University of Texas at Dallas, UNITED STATES

## Abstract

**Objective:**

Repetitive Transcranial Magnetic Stimulation (rTMS) is a novel therapeutic tool to induce a suppression of tinnitus. However, the optimal target sites are unknown. We aimed to determine whether low-frequency rTMS induced lasting suppression of tinnitus by decreasing neural activity in the cortex, navigated by high-density electroencephalogram (EEG) source analysis, and the utility of EEG for targeting treatment.

**Methods:**

In this controlled three-armed trial, seven normal hearing patients with tonal tinnitus received a 10-day course of 1-Hz rTMS to the cortex, navigated by high-density EEG source analysis, to the left temporoparietal cortex region, and to the left temporoparietal with sham stimulation. The Tinnitus handicap inventory (THI) and a visual analog scale (VAS) were used to assess tinnitus severity and loudness. Measurements were taken before, and immediately, 2 weeks, and 4 weeks after the end of the interventions.

**Results:**

Low-frequency rTMS decreased tinnitus significantly after active, but not sham, treatment. Responders in the EEG source analysis-based rTMS group, 71.4% (5/7) patients, experienced a significant reduction in tinnitus loudness, as evidenced by VAS scores. The target site of neuronal generators most consistently associated with a positive response was the frontal lobe in the right hemisphere, sourced using high-density EEG equipment, in the tinnitus patients. After left temporoparietal rTMS stimulation, 42.8% (3/7) patients experienced a decrease in tinnitus loudness.

**Conclusions:**

Active EEG source analysis based rTMS resulted in significant suppression in tinnitus loudness, showing the superiority of neuronavigation-guided coil positioning in dealing with tinnitus. Non-auditory areas should be considered in the pathophysiology of tinnitus. This knowledge in turn can contribute to investigate the pathophysiology of tinnitus.

## Introduction

Subjective tinnitus is characterized by the perception of a phantom sound (e.g., ringing) experienced in the absence of any external acoustic stimulus. This disturbing phenomenon is experienced by 10–15% of the general population, and it significantly impacts the quality of life in about 20% of the adults experiencing tinnitus, who will require clinical intervention [1].

Subjective tinnitus is often accompanied by peripheral hearing loss; thus, it has been thought for many years that the tinnitus-related neural activity must be originate from a peripheral source, such as the cochlea [2]. However, some clinical observations indicate that a peripheral origin of tinnitus does not account for all sources of tinnitus. Indeed, in an effort to relieve tinnitus, sectioning of the eighth cranial nerve has been performed in tinnitus patients. However, this was unsuccessful in the management of tinnitus in 85% of cases [3, 4]. Clearly, tinnitus may arise even when disconnecting the cochlea from the brain.

Thus, central mechanisms must be responsible for most tinnitus. For many forms of subjective tinnitus, mechanisms in the central nervous system have long been sought in pathology. Specifically, three central mechanisms have been proposed to underlie tinnitus: (1) changes in the firing pattern of neurons in the central auditory system, (2) changes in burst firing and neural synchrony, and (3) cortical tonotopic map reorganization [5]. All of these may occur as a consequence of alterations of neuronal activity involving both the auditory system and non-auditory areas, which may underlie a potentially promising treatment strategy to tinnitus.

It is difficult to treat chronic refractory tinnitus. Drugs or cognitive-behavioral therapy that attempt to improve the tinnitus have been tried for many years. Unfortunately, the success rate remains low [6]. Recently, a promising strategy, repetitive transcranial magnetic stimulation (rTMS) was reported to induce lasting suppression of tinnitus. rTMS is a novel diagnostic and therapeutic tool, targeting hyperactivity/abnormal synchronization for a variety of psychological and neurological conditions. rTMS temporarily produces disruptions in a circumspect area of the cortex that interrupts normal functioning and has acute and chronic effects [7]. However, the optimal target sites, as well as the most favorable treatment schedules remain unknown. It is difficult to navigate the burst firing in the central auditory system, which may decrease the treatment effects because of methodological weaknesses [8].

Functional magnetic resonance imaging (fMRI) and positron emission tomography (PET) are imaging modalities that can be used to study neural activity in the human brain. However, potential limitations of PET and fMRI may preclude their widespread application. Using radioactive tracers in PET, ionization is induced in the human body, making it less suitable for repeated measurements of single subjects. In fMRI, a major limitation, especially in auditory research, is simply the acoustic noise produced by the scanner. During scanning, the MR scanner typically produces over 100 dB (SPL) of noise, making it difficult to segregate responses to testing auditory stimuli from those to ambient scanner noise [5].

More recently, investigations using high-density electroencephalography (EEG) recordings with a high degree of spatiotemporal resolution, in combination with source analysis, have been reported [9]. Functional changes in auditory cortex activity can be revealed in tinnitus patients using high-density EEG. EEG is a non-invasive method that measures electrical and magnetic fields, resulting from the firing of neurons.

Thus, EEG gives rise to an opportunity for the optimization of treatment strategies. In humans, the different localization of neuronal generators in source analysis of EEG signals in subjects with tinnitus compared with control subjects and the efficacy of EEG source analysis-based rTMS for the treatment of tinnitus have not been reported previously.

In this pilot study, we sought to explore the different localization of neuronal generators in source analysis of EEG signals in subjects with tinnitus versus control subjects. Also, for the first time, we assessed the efficacy of rTMS navigated with high-density EEG source analysis on tinnitus using various clinical scales.

## Methods

### Study Patients

After approval by Shanghai Jiao Tong University Human Subjects Review Committee, written informed consent was obtained from all subjects before the study and possible consequences of the study were explained.

In total, 21 right-handed subjective tinnitus patients were divided into three groups, seven in each. Seven right-handed subjective tinnitus patients with tonal tinnitus and without hearing loss were recruited into group A. These patients with tonal tinnitus at a frequency of 2,000 Hz underwent rTMS navigated with EEG GeoSource. Seven subjective tinnitus patients with tonal tinnitus and without hearing loss were recruited into group B. The patients with tonal tinnitus underwent rTMS over the left temporoparietal cortex region. Seven subjective tinnitus patients with tonal tinnitus and without hearing loss were recruited into group C, and underwent left temporoparietal sham stimulation. Also, 10 right-handed volunteers with normal hearing and without tinnitus participated as controls, in group D. The tinnitus subjects and controls were of comparable age and gender. The sham stimulation, as described in the Methods section, looks and sounds like the active coil, but diverts the magnetic field away from the patient. Data were collected regarding the patients’ gender, age, tinnitus type, related ear, tinnitus pitch, tinnitus loudness, and tinnitus duration. Patients with noisiform tinnitus were excluded.

Upon recruitment, patients answered a baseline tinnitus rating questionnaire prior to treatment. The Tinnitus handicap inventory (THI) has been used widely to scale tinnitus severity. The 25 items are grouped into functional, emotional, and catastrophic subscales. Additionally, the subjective tinnitus loudness perception in patients was obtained with a visual analog scale (VAS) from 0 to 10, where 0 means no tinnitus and 10 means the worst possible tinnitus-related discomfort. Measurements were taken before, and immediately, 2 weeks, and 4 weeks after the end of the interventions.

### Auditory testing and tinnitus matching

Hearing level and tinnitus frequency was assessed with an audiogram. The patients and the volunteers who had normal audiometry (threshold below 25 dB HL at all frequencies from 0.25 to 8 kHz), normal tympanometric curve of type A, and presented ipsi- and contralateral stapedial reflexes were included. The intensities and frequencies of the tinnitus were evaluated with a TinniTest audiometer in a sound-proof booth in unilateral or bilateral tinnitus patients. The participants were asked to concentrate on the predominant pitch of the tinnitus, and then the tinnitus sound was identified by matching it to the sounds presented.

### 256-channel high-density EEG recordings

For the EEG recording, subjects were requested to refrain from alcohol or caffeinated beverages consumption 24 h prior to and on the day of recording. EEG data were recorded with 256 channels on EGI’s HydroCel Geodesic Sensor Net and Cz was used as the reference channel. Each subject underwent ~30 min of recordings with a dense-array scalp EEG technique. The 256-channel Geodesic Sensor Net was applied to the subjects before the recording, requiring about 10 min for adjustment until the impedances were checked to remain below 50 kΩ. The odd-ball stimulus pattern with pure tone (1000–2000 Hz, 75 dB, 50-ms duration with a shaped 5-ms rise and fall time) was used (via Eprime 2.0, Psychology Software Tools). A regular stimulus of 1000 Hz was presented in 85% of the trials, together with a deviant stimulus according to the frequency of tinnitus at 2000 Hz in 15% of the trials. The whole task consisted of a total of 1000 auditory stimuli with random inter-stimulus intervals (ISI), ranging from 850 to 1450 ms. Sound stimuli with an intensity of 75 dB were delivered through two loudspeakers at a distance of 100 cm from the subjects. The two loudspeakers were positioned 45° to the ears and 90° to each other. Subjects sat on a comfortable chair in a sound-proof booth, watching silent films they were interested in. They were not asked to respond to any auditory stimulus during the test. At the end of each session, all subjects reported that they were awake during the whole experiment and paid attention to the film. All testing was done in the morning to avoid the subjects feeling sleepy in the afternoon or evening. Source analysis of event-related potentials as mismatch negativity (MMN), describing the neural sources of the measured scalp potentials, was estimated using GeoSource (ver. 2.0).

### rTMS procedure

Repetitive TMS consisted of 1000 stimulations/day at 1 Hz and 110% of the motor cortex threshold for 5 consecutive days (Monday to Friday) per week and lasted 2 weeks, using the same coil and stimulator. For repetitive pulses, TMS was delivered through a focal figure-of-eight magnetic coil connected to a magnetic stimulator (MagPro R30, MagVenture A/S, Denmark). With the EEG system as the targeting reference, the location of the maximum intensity determined by the sources analysis of the difference between standard and deviant stimuli in MMN processing was used for the target in rTMS stimulation. The sham stimulation looks and sounds like the active coil, but diverts the magnetic field away from the patient.

### Data analysis

EEG data were recorded continuously at a sampling rate of 250 Hz. Data processing was carried out using Net Station (EGI, USA). The continuous EEG data were filtered digitally (0.1–30 Hz). The segmentation of the EEG was excluded if it was contaminated by ocular artifacts (e.g., blinks) or if it contained 10 or more channels of data that exceeded a voltage threshold of 200 mV or a transition threshold of 100 mV. After averaging, the data were re-referenced to the average reference and baseline-corrected. Finally, the trials from each subject were grandly averaged. The difference waveform between deviant and standard stimuli was also calculated to determine the components of MMN. MMN was the difference waveform between deviant and standard stimuli, which was identified as a negative component between 100 and 250 ms after subtracting the deviant waveform from the standard waveform. Source analysis, describing the neural sources of the measured scalp potentials, was estimated with GeoSource (ver. 2.0). Standardized low-resolution brain electromagnetic tomography (sLORETA) was performed to evaluate the brain sources for the MMN components versus the grand average of the tinnitus patients. The maximum intensity, indicating the most active area in the whole brain in the tinnitus group was navigated to in the TMS procedure.

Repeated-measures analysis of variance (ANOVA) was used for analysis of THI and VAS scores. The main analysis of this study was to evaluate the impact of rTMS on the perception of tinnitus navigated by EEG source analysis. The primary efficacy measure was defined as the decrease in the THI score and VAS scores. Statistical significance was set at a p-value ≤ 0.05. In this double-blinded study, neither the subjects nor the medical personnel assessing the effects of the intervention knew which subjects received the active and sham stimulation. The statistician and the medical monitor held the sealed code and during the study nobody was to break the code except for an emergency. The code was unsealed only after the study was finished and the statistical analysis was complete.

## Results

Seven patients with tonal tinnitus at a frequency of 2,000 Hz underwent rTMS navigated by EEG source analysis in group A (age range: 34–62 years, mean 51.85 years; 3 men, 4 women). Seven patients with tonal tinnitus and without hearing loss underwent rTMS over the left temporoparietal cortex region, regardless of tinnitus laterality in group B (age range: 40–63 years, mean 53.42 years; 2 men, 5 women). Seven normal hearing patients (age range: 42-62 years, median age of 52.43 years) with tonal tinnitus underwent left temporoparietal sham stimulation in group C. Chronic tinnitus in all participants was of 0.8–11 years in duration. Ten right-handed volunteers (age range: 36–60 years, mean 52.2 years; 3 men, 4 women) with normal hearing and without tinnitus were recruited as controls, in group D. The clinical characteristics of the tinnitus patients are shown in Table 1. All the patients tolerated rTMS well and no adverse effect was observed. There was no significant difference in age between the three groups (p > 0.05). There was no significant difference of tinnitus duration between the three groups.

**Table 1:** Clinical characteristics of tinnitus patients.

Group	NO	Age	Gender	Side of tinnitus	Duration of tinnitus	Pitch of tinnitus	Loudness of tinnitus	Hearing threshold at tinnitus frequency
Group A	NO1	57	Male	bilateral	9 months	pure tone 2000 Hz	10 dB	5 dB
	NO2	62	Female	bilateral	9 years	pure tone 2000 Hz	19 dB	10 dB
	NO3	40	Female	right	3 years	pure tone 2000 Hz	41 dB	25 dB
	NO4	55	Male	bilateral	6 years	pure tone 2000 Hz	25 dB	10 dB
	NO5	34	Female	left	3 years	pure tone 2000 Hz	38 dB	25 dB
	NO6	58	Male	bilateral	10 years	pure tone 2000 Hz	25 dB	20 dB
	NO7	57	Female	right	4 years	pure tone 2000 Hz	32 dB	20 dB
Group B	NO1	40	Male	bilateral	8 months	pure tone 750 Hz	33 dB	25 dB
	NO2	63	Male	bilateral	5 years	pure tone 2000 Hz	30 dB	25 dB
	NO3	55	Female	left	9 months	pure tone 1500 Hz	15 dB	5 dB
	NO4	60	Female	bilateral	2 years	pure tone 3000 Hz	43 dB	25 dB
	NO5	57	Female	right	1 years	pure tone 8000 Hz	25 dB	15 dB
	NO6	48	Female	right	3 years	pure tone 4000 Hz	30 dB	20 dB
	NO7	51	Female	left	8 months	pure tone 1500 Hz	35 dB	25 dB
Group C	NO1	42	Male	bilateral	3 years	pure tone 8000 Hz	15 dB	5 dB
	NO2	50	Female	right	10 months	pure tone 6000 Hz	37 dB	20 dB
	NO3	58	Male	bilateral	1 years	pure tone 1500 Hz	33 dB	25 dB
	NO4	62	Male	bilateral	11years	pure tone 4000 Hz	29 dB	25 dB
	NO5	56	Female	left	2 years	pure tone 2000 Hz	28 dB	15 dB
	NO6	55	Female	bilateral	3 years	pure tone 1000 Hz	35 dB	25 dB
	NO7	44	Female	left	9 months	pure tone 1500 Hz	30 dB	20 dB

**Fig 1:**
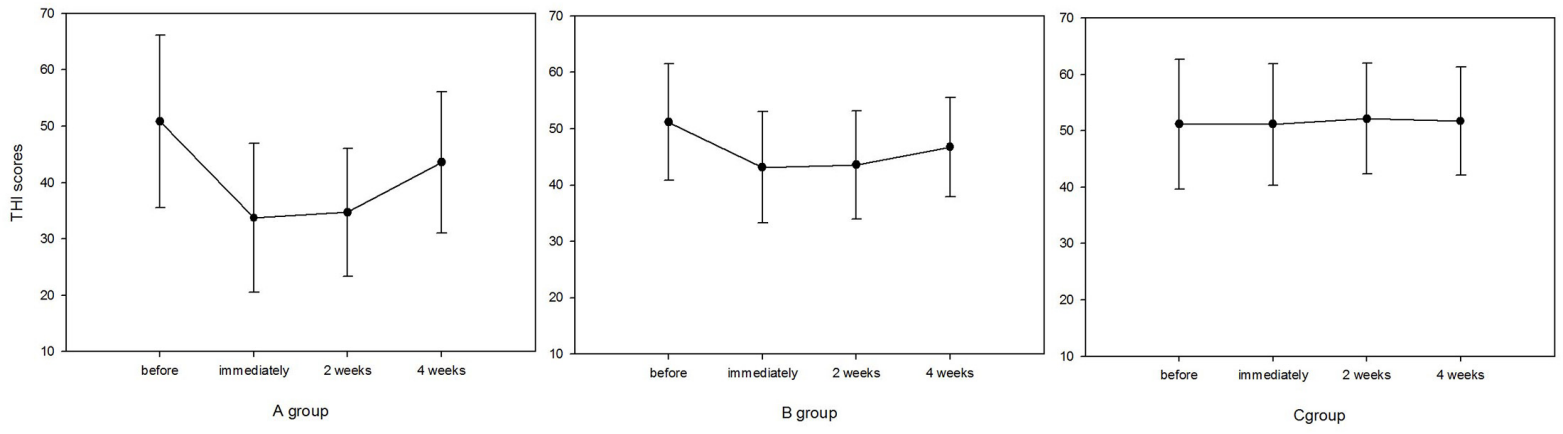
Changes in mean THI scores from baseline to 4 weeks after rTMS treatment. Error bars indicate standard deviations.

The baseline differences between groups regarding THI scores were insignificant (F = 0.132, P = 0.877). Repeated measures ANOVA was performed taking time and intervention as within and between-subject factors, respectively, revealed a significant main effect of time (F = 8.936, P<0.001) and intervention (F = 81.832, P<0.001). A post hoc evaluation was performed within the factor of time after stimulation to verify the significance of the scores at different time points. It was found that the THI scores were decreased significantly immediately after the intervention compared with baseline in A group, whereas 4 weeks later, there was no significant difference in THI scores versus baseline (Fig 1).

Responders in the EEG source analysis-based rTMS group, 71.4% (5/7) of patients, experienced a significant reduction in tinnitus loudness, as evidenced by THI and VAS scores. Also, 42.8% (3/7) patients experienced a decrease in tinnitus loudness after left temporoparietal rTMS stimulation. In the sham stimulation group, there was no significant change in THI or VAS scores after the intervention.

For the subjects who underwent the EEG source analysis-based rTMS, active treatment was associated with an average reduction of 11.7 (range, 2-31) points in THI scores, and non-EEG-based TMS was associated with a median reduction of 7.57 (range, -2 to 25) points. However, in group C, with sham stimulation, there were no significant changes in THI scores after the intervention.

**Fig 2:**
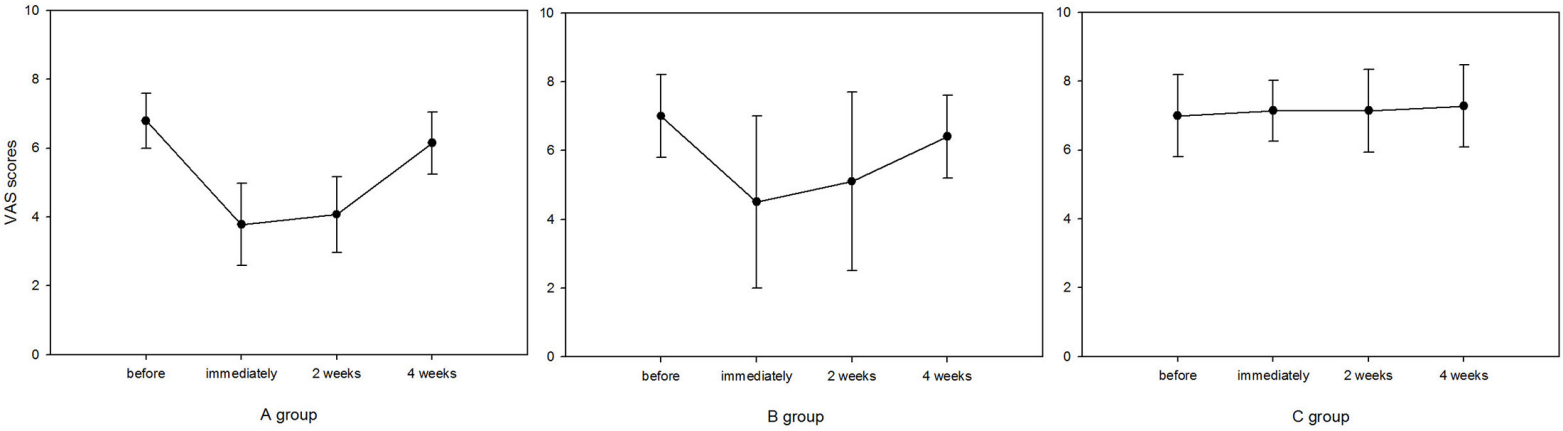
Changes in mean VAS scores from baseline to 4 weeks after rTMS treatment. Error bars indicate standard deviations.

Before the study, the baseline mean VAS score in groups A, B, and C were 6.14 (4–8), 5.85 (3–8), and 5.28 (3–8), respectively. The average VAS scores immediately after active and sham interventions in groups A, B, and C were 4.7 (3–8), 5.0 (3–8), and 5.4 (3–8), respectively (Fig 2). There were 2 (28.6%) subjects who achieved a half-of-the-baseline decrease or larger reduction in group A and 1 (14.3%) subject in group B. The baseline differences between groups regarding VAS were insignificant (F = 0.04, P = 0.96). Repeated measures ANOVA was performed taking time and intervention as within and between-subject factors, respectively, revealed a significant main effect of time (F = 13.693, P<0.001) and intervention (F = 222.863, P<0.001). In group A post hoc testing for different time points revealed significant differences between baseline and immediately after the intervention (P = 0.01), and between baseline and 2 weeks after the intervention (P = 0.01). In group B, post hoc testing revealed insignificant differences between baseline and immediately after the intervention and between baseline and 2 weeks after the intervention (P = 0.079 and P = 0.116, respectively).

As indicated in previous studies, the presence of normal hearing and distortion-product otoacoustic emissions (DPOAE) was positively associated with a significant effect in rTMS treatment. The patients with bilateral tinnitus more often reported a reduction of tinnitus than those with unilateral tinnitus after the rTMS procedure. However, this effect in some patients declined over time.

### EEG results

**Fig 3:**
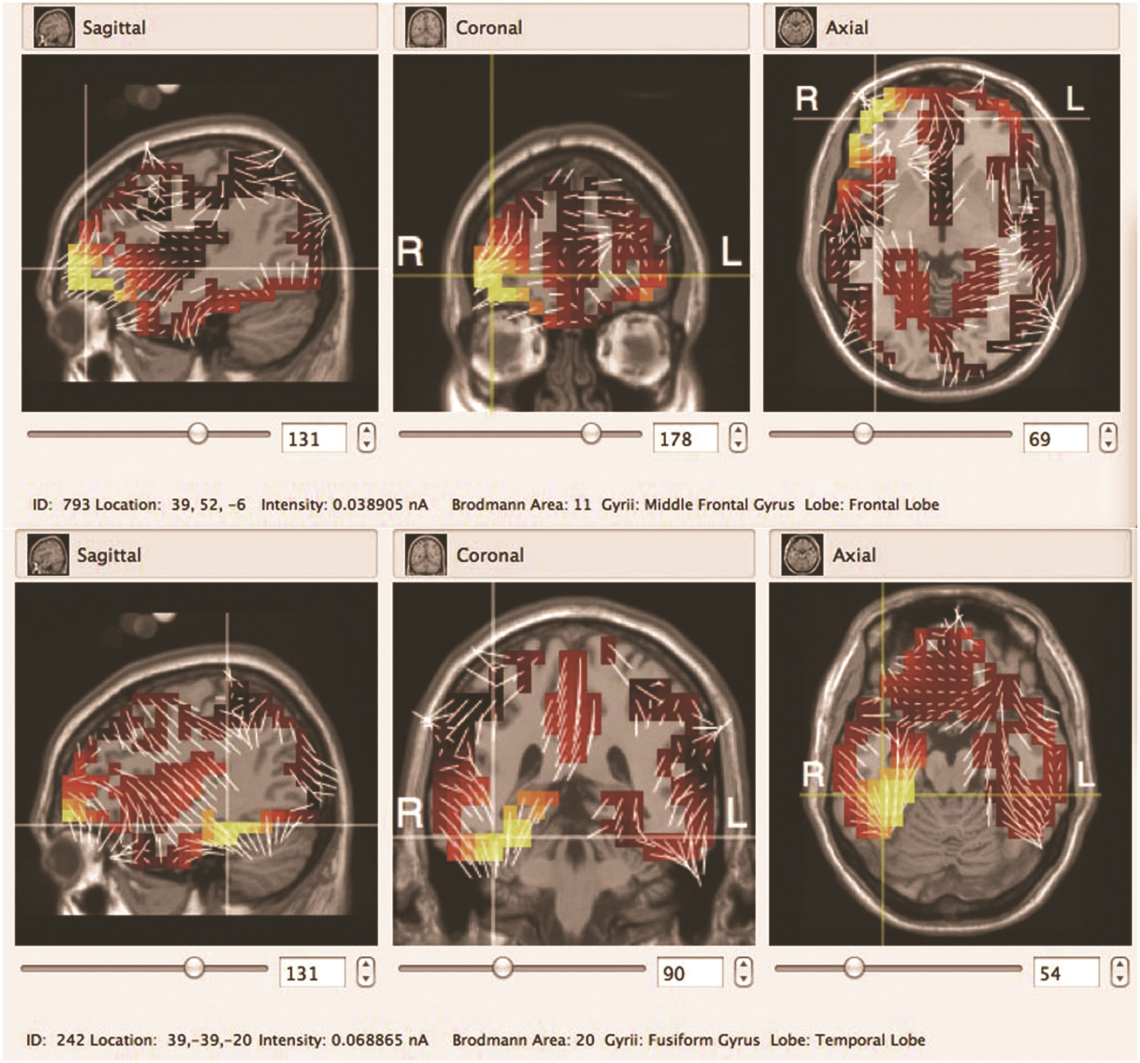
Source model for the difference of standard stimuli and deviant stimuli (the frequency of tinnitus): tinnitus group (A) and control group (B). The intensity of the topmost dipole is located in the frontal lobe in Brodmann Area 11 in the tinnitus group. The intensity of the topmost dipole is located in the temporal lobe in Brodmann Area 20 in the control group.

**Fig 4:**
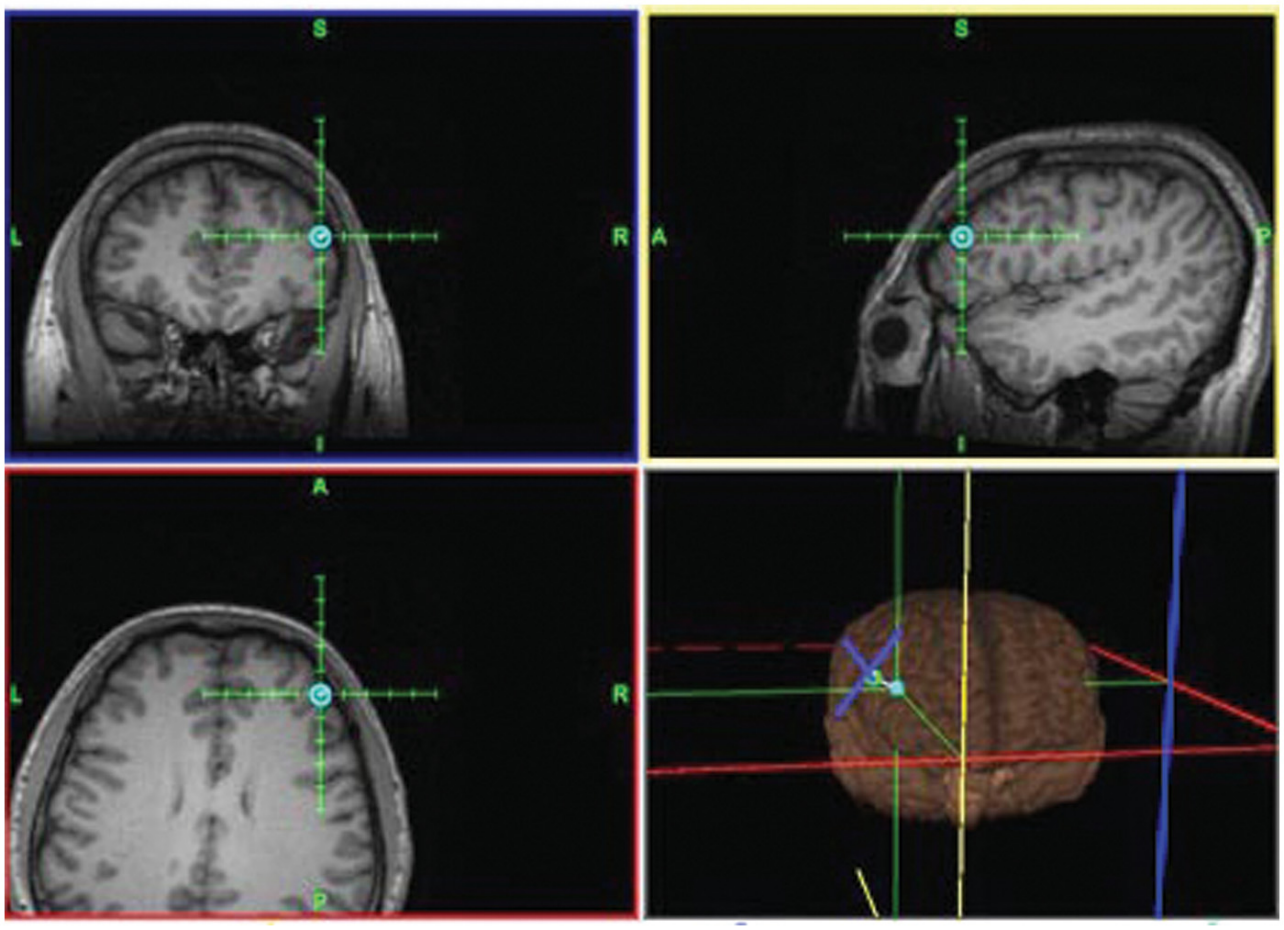
Location of the targets. The location of the EEG-maximum intensity in the cortex, identified by source analysis, is presented for one patient, as defined using the image-guided neuronavigation system.

**Fig 5:**
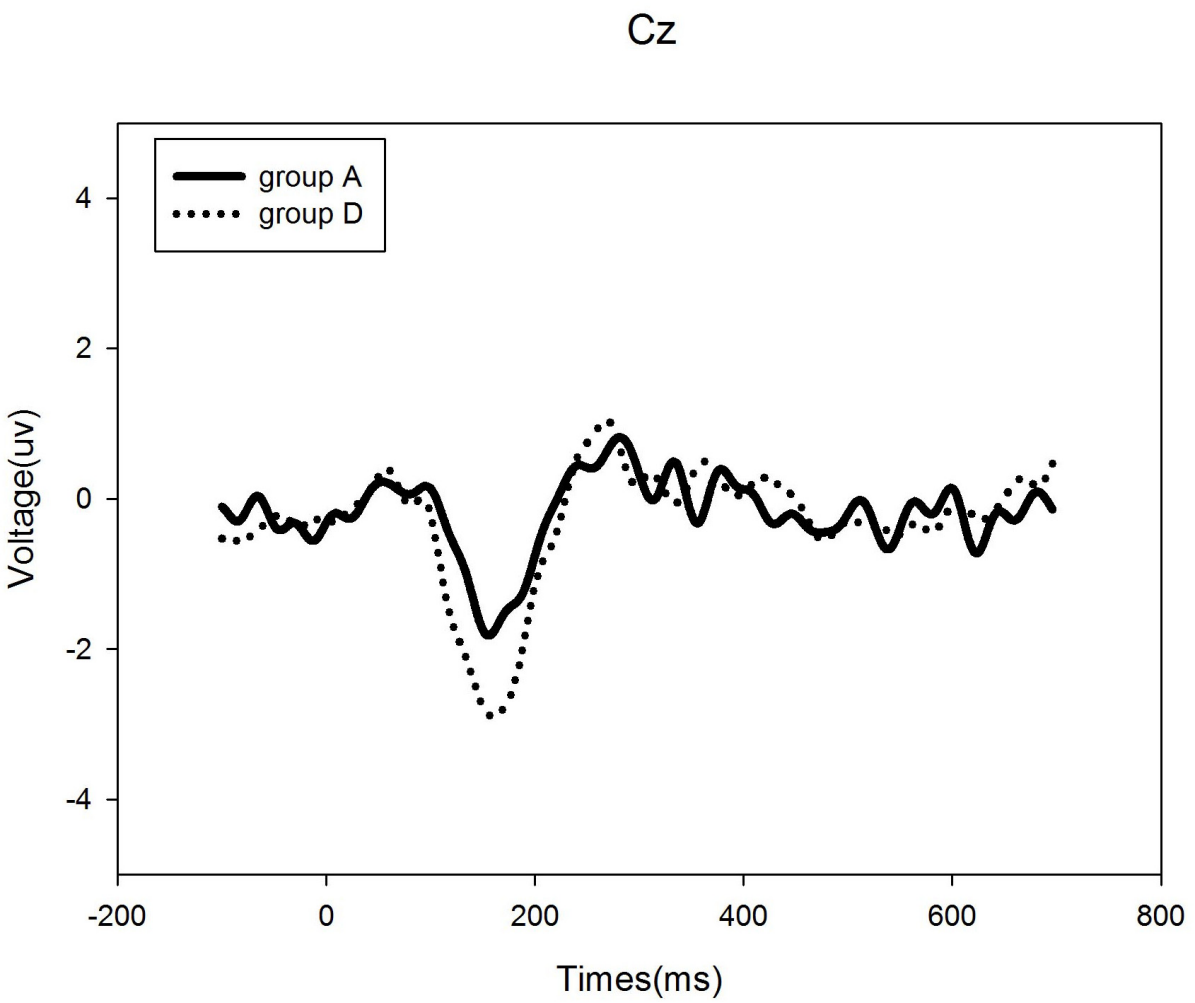
Grand-averaged ERPs to the standard and deviant stimuli for tinnitus group (group A) and control group (group D) at Cz. The solid thick line illustrates the ERP to deviant stimuli in group D, and the dashed thick line, the ERP to the deviant stimuli in group A.

We found that patients with tonal tinnitus exhibited specific patterns of cortical activities, which differed from healthy control subjects, as evidenced by the source analysis of the components of MMN. As illustrated with the GeoSource Viewer (Fig 3), dipole intensities are displayed, overlaid on sagittal, coronal, and axial MRI slices in the control group and tinnitus patients. What is seen is a reorganization of the difference responses to the deviant stimuli at the right frontal lobe in the tinnitus patients. The intensity of the topmost dipole is located in the frontal lobe, in Brodmann Area 11. The number dipole of 600 in the tinnitus subjects has values 39, 52, and -6 for *x*, *y*, and *z* coordinates, respectively. However, the source model in the temporal lobe in the control group has values of 39, -39, and -20 as *x*, *y*, and *z* coordinates, respectively, in Brodmann Area 20. 1 Hz rTMS for 10 days was applied in tinnitus patients at a frequency of 2000 Hz, navigated to the EEG maximum intensity in the cortex, as identified by source analysis (Fig 4). With the frequency of tinnitus as a deviant stimulus, the between-group comparison of the MMN amplitude revealed a significant difference. Tinnitus patients in group A showed a decreased MMN amplitude compared with control subjects in group D at Cz (p < 0.05). There was no significant difference in MMN latency in group A versus group D (p > 0.05; Fig 5).

## Discussion

The present study demonstrated that active EEG source analysis-based rTMS resulted in significant suppression of tinnitus loudness, and supports the hypothesis that changes in tinnitus perception correlate significantly with the activity in regions of interest sourced by EEG. Low-frequency rTMS improves tinnitus by reducing cortical activation at the stimulation site, confirming the utility of EEG for targeting rTMS. However, the efficacy of tinnitus reduction in some patients declined over time. Non-auditory areas have been considered to be involved in the pathophysiology of tinnitus. This knowledge, in turn, may contribute to investigating the pathophysiology of tinnitus.

Many efforts have been made to gain insights into the neurophysiological mechanisms of tinnitus [10]. A consensus has been reached that this phantom perception may be attributable to an abnormal neural signal, such as burst-firing activity or neural synchrony [11, 12]. Both positron emission tomography (PET) and functional magnetic resonance imaging (fMRI) are based on the support that a change of neuronal activity alters the local metabolism and perfusion of the brain. PET mainly measures a change in regional cerebral blood flow, while most fMRI methods register a blood oxygen level dependent signal. Evidence for changes in the firing pattern of neurons in the central auditory system as a possible substrate of tinnitus might be assumed to be supported by the findings of positron emission tomography (PET) and functional magnetic resonance imaging (fMRI) studies in humans [5, 13]. Moreover, tinnitus with loss of input caused by peripheral hearing loss is accompanied by changes in the tonotopic map in the auditory cortex.[14]. The most important information obtained from imaging of cerebral function was the location, the extent, and the magnitude of neural activity. Thus, the question that may be addressed by the application of imaging methods of cerebral function is which brain regions have an abnormal amount of neural activity in tinnitus subjects?

Obviously, target identification is essential for the use of focal brain stimulation. Target selection has to be based on the best available knowledge of the neuronal correlate of the disorder. fMRI and PET have been used to study neural activity in the human brain. Several studies using PET and fMRI scans have recognized areas of excessive asymmetric cortical activity in the left or right temporal cortex [8, 15, 16]. However, using radioactive tracers in PET, ionization is induced in the human body, making it less suitable for repeated measurements of single subjects, which may prelude some studies. During fMRI experiments, in addition to contraindications for MRI research in humans, the intensity of the acoustic noise produced by the scanner is typically over 100 dB (SPL), which would disturb any auditory stimuli, making it difficult to segregate responses from task-related changes in neural activity. More recently, EEG in combination with source analysis has provided non-invasive assessments of cortical activity [9]. Moreover, functional changes in the cortex associated with auditory activity can be revealed in tinnitus patients using high-resolution EEG. Source analysis of event-related potentials (ERPs) is a sensitive method to assess the changes evoked by tinnitus in the central nervous system [17]. After tinnitus matching, as the frequency of tinnitus was used to deviant stimuli, active areas in the brain in the difference between standard stimuli and deviant stimuli can be recognized in the tinnitus group and the normal control group by EEG source analysis. Regional source models superimposed on magnetic resonance images of each subject were displayed on sagittal, coronal, and axial MRI slices. This provides an opportunity to optimize the brain regions associated with abnormal amounts of neural activity in tinnitus subjects. In our study, alterations in neuronal activity in tinnitus subjects were not limited to the auditory system but also involved non-auditory brain areas, such as the frontal lobe, consistent with previous studies [18–20]. These non-auditory areas may indicate potentially promising treatment strategies for tinnitus.

rTMS allows focal stimulation of the cortex and, accordingly, has been shown to be able to decrease tinnitus, even if the exact mechanisms of action of the different applications remain unclear. The magnetic field passes through the skull and induces a small secondary current in the cortex, with resulting inhibition or excitation, depending on the frequency of stimulation. It has been assumed that low-frequency rTMS exerts its effects by inducing long-term depression-like effects and high-frequency rTMS can be used to enhance cortical activity [21]. An alternative explanation is that rTMS over the auditory cortex disrupts connectivity between the auditory system and non-auditory areas involved in tinnitus generation and thereby facilitates the intrinsic ability of the brain to restore normal function [22]. Evidence from previous studies suggested that tinnitus causes consistent modifications to functional networks, including greater connectivity between limbic areas and cortical networks not traditionally considered to be involved with emotion processing, and increased connectivity between attention and auditory processing brain regions [23, 24]. This may further explain why after stimulation over non-auditory areas, such as the frontal lobe sourced by EEG in our study, rTMS induced superior effects in tinnitus reduction. Unlike the available applicable strategies, such as the 10–20 EEG system, EEG source analysis-based rTMS provides clear superiority for neuronavigation-guided coil positioning in dealing with tinnitus. This knowledge may, in turn, contribute to further investigations on the pathophysiology of tinnitus.

Subgrouping of tinnitus patients based on tinnitus characteristics or imaging results may contribute to developing more individualized treatment protocols. In our study, we focused on normal hearing patients with chronic tonal tinnitus, where it is assumed that tinnitus as an auditory percept is due mainly to changes in the central auditory system. Patients with narrowband noise tinnitus were excluded from this study.

In conclusion, using rTMS in combination with electrophysiological techniques, such as EEG, may contribute to identifying the neurobiological mechanisms of tinnitus with optimization of the role of stimulation laterality, the exact coil position, and coil orientation. Non-auditory areas have been considered to be involved in the pathophysiology of tinnitus, as evidenced by the available functional imaging data. Their potential influence on tinnitus and individualized treatment protocols should be further investigated in subgroups of distressed or chronic tinnitus patients.

## Supporting Information

S1 Data Data for this meta-analysis. (XLSX)
